# Cultural Differences in Older Immigrants' Health and Social Services Use

**DOI:** 10.1093/geroni/igab046.1658

**Published:** 2021-12-17

**Authors:** Freya Diederich

**Affiliations:** University of Bremen, Bremen, Bremen, Germany

## Abstract

Even though Germany has a mandatory health and long-term care insurance with no or only very low co-payments, immigrants and the native population differ in their health and social services use. Differences in cultural traits and a lack of knowledge about the institutional setting are frequently mentioned as contributing factors. Relying on the epidemiological approach in the economic literature, this empirical study shows that both cultural traits that prevail in older immigrants’ country of origin and older immigrants’ knowledge about the host country’s institutional setting affect their health and social services use in Germany. We distinguish foreign-born immigrants and their descendants as both groups differ in their connection to the home and the host country. The results will be used to discuss immigrants’ access and potential barriers to the use of health and social services in comparison to the native population.

